# Stress cardiomyopathy in the paediatric population: a case series

**DOI:** 10.1093/ehjcr/ytae030

**Published:** 2024-01-29

**Authors:** Nadine Annino, Aymeric Cantais, Etienne Javouhey, Florent Baudin

**Affiliations:** Paediatric Emergency Department, Hospital University of Saint Etienne, Hôpital Nord, 42055 Saint Etienne Cedex 2, France; Paediatric Emergency Department, Hospital University of Saint Etienne, Hôpital Nord, 42055 Saint Etienne Cedex 2, France; Paediatric Intensive Care Unit, Hopital Femme Mère Enfant, Hospices Civils de Lyon, University of Lyon, Bron, France; Paediatric Intensive Care Unit, Hopital Femme Mère Enfant, Hospices Civils de Lyon, University of Lyon, Bron, France; Agressions Pulmonaires et Circulatoires dans le Sepsis (APCSe), VetAgro Sup, Universités de Lyon, Marcy l’Etoile, France

**Keywords:** Stress cardiomyopathy, Paediatric, Case series

## Abstract

**Background:**

Stress cardiomyopathy (Takotsubo syndrome) defined as Takotsubo syndrome is defined as a reversible acute myocardial syndrome with myocardial injury with regional wall motion abnormality and no coronary explanations in the context of stress. The pathophysiology remains partially unknown, and these cases are probably underestimated in paediatrics. We report six cases of Takotsubo probably secondary to neurological damage.

**Case summary:**

Six patients (10, 13, 16, 10, and 9 years and 5 months) presented with haemodynamic lability with echocardiography data leading to suspicion of Takotsubo syndrome. These cases were secondary to neurological involvement (cerebral haemorrhage, intraventricular haemorrhage, brain damage due to bifrontal oedema, posterior fossa tumour, pneumococcal meningitis, high-grade glioma). All patients were rapidly started on amine. Reversibility of the acute myocardial syndrome was complete in all but one child, who rapidly progressed to encephalic death.

**Discussion:**

Neurological distress has been suggested as a potential cause of Takotsubo syndrome. The pathophysiology is possibly related to excessive stimulation of the sympathetic system. This syndrome should probably be considered in the setting of left heart failure with neurological distress so as not to delay the use of amines especially since in the paediatric population the probability of a coronary origin is low.

**Learning points**

- Takotsubo is not uncommon in the paediatric population, and the trigger is basically neurologic.
- Stress cardiomyopathy may be considered a gravity factor in neurologic disease and must be systematically tracked.

## Introduction

Stress cardiomyopathies are acute conditions characterized by left ventricular (LV) dysfunctions that occur independently of coronary issues, often triggered by intense emotional or physical stress.^1^ The most well-known variant is Takotsubo syndrome, first identified by Sato in Japan in 1990.^2^ It’s typified by transient LV dysfunction, predominantly affecting the apex.^3^ The term ‘Takotsubo’ is derived from a Japanese octopus trap whose shape resembles the LV’s characteristic apical ballooning during systole. While the exact pathophysiology is still not fully understood, initial theories suggest sympathetic hyperstimulation and elevated circulating catecholamines as primary factors.^4,5^

The diagnosis of Takotsubo syndrome hinges on four criteria^6–8^: (i) transient LV dysfunction with contractility disturbances and abnormal LV wall movement at the apical or mid-ventricular level, not confined to a coronary territory; (ii) absence of coronary anomalies (coronary angiographies are rare in children, and acute myocardial syndromes are also uncommon; the decision to forego coronary assessment is debatable); (iii) recent non-specific electrocardiogram (ECG) abnormalities, like ST-segment elevation or T-wave inversion, or moderate elevation of cardiac enzymes; and (iv) absence of pheochromocytoma or myocarditis. In 2018, an expert panel updated these criteria to include neurological conditions and pheochromocytoma as potential triggers and acknowledged that coronary anomalies might be associated with the syndrome.^9,10^ The 2023 European Resuscitation Council (ERC) guidelines for acute coronary syndrome management include Takotsubo as part of the initial evaluation for myocardial infarction with non-obstructive coronary arteries after coronary angiography.

In children, stress cardiomyopathies are likely underdiagnosed and present differently compared with adults.^11^ They affect both sexes equally,^12^ often involve frustrating initial symptoms like consciousness disturbances or heart failure signs (such as nausea or vomiting), and are associated with a higher incidence of ST-shift on ECG. Younger patients tend to have more non-apical forms and severe LV dysfunction.^12^ While stress cardiomyopathy can occur at any age in childhood, it is less common in infants.^13^

There is limited data on paediatric stress cardiomyopathy characteristics, but it is relatively prevalent, especially in severe acute neurological diseases.^14,15^ Our aim is to describe all cases of paediatric stress cardiomyopathy admitted to our paediatric intensive care unit (PICU) from January 2015 to July 2018, regardless of cause, to better understand the circumstances and clinical situations in which this syndrome manifests in children.

## Summary figure

**Figure:**
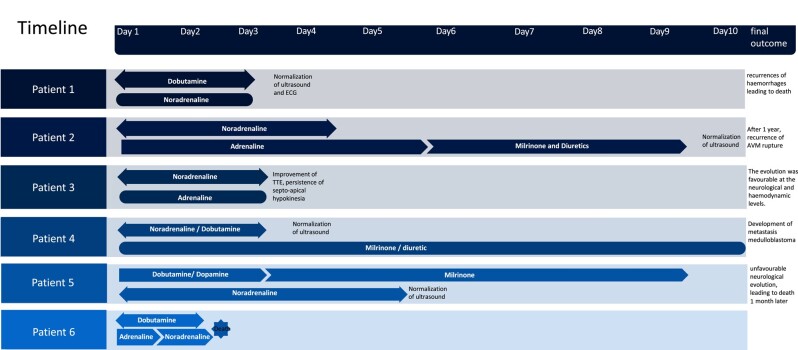


## Case summaries

### Patient 1

A 10-year-old Caucasian girl, weighing 25 kg and with a history of cavernoma, was admitted to the PICU due to a recurrent brain haemorrhage, indicated by severe headache and oculomotor disorders. A brain computed tomography (CT) scan suggested possible brainstem infiltration and compression of the fourth ventricle. Upon admission, she exhibited tachycardia but no initial haemodynamic failure, and her Glasgow Coma Scale score was 15.

She developed haemodynamic failure and required support with dobutamine up to 5 µg/kg/min for 48 h, along with noradrenaline up to 0.2 µg/kg/min, also for 48 h. The ECG revealed pathological changes including diffuse ST-segment elevation, QS block, sinus tachycardia, and a right axis deviation.

Transthoracic echocardiography (TTE) showed Takotsubo-type stress cardiomyopathy with segmental dysfunction of the LV involving the apex, characterized by a septal bulge and an acceleration to 4 m/s exacerbated by tachycardia. The LV ejection fraction (LVEF) was 33%, but cardiac output remained preserved. Apart from these changes, the cardiac architecture was normal. Troponin levels peaked at 650 ng/L before decreasing.

Left ventricular function began improving from Day 4, accompanied by enhancements in ultrasound appearance and normalization of the ECG. Her condition was further complicated by multiple recurrences of peduncular, pontine, cerebellar, and meningeal cerebral haemorrhages, ultimately leading to her death.

### Patient 2

A 13-year-old Caucasian girl, weighing 45 kg and with no prior medical history, was admitted to the PICU for acute intracranial hypertension resulting from tetra ventricular bleeding caused by a ruptured deep right arteriovenous malformation (AVM). At admission, she exhibited high heart rate variability and had a Glasgow Coma Scale score of 3 without sedation. During her initial neurosurgical procedure, she experienced a cardiocirculatory arrest but was revived after receiving five doses of adrenaline.

Following this, she showed haemodynamic instability and required support with dobutamine at 20 µg/kg/min (weaned on Day 9), noradrenaline at 0.8 µg/kg/min (weaned on Day 4), and adrenaline at 0.5 µg/kg/min (weaned on Day 5). The use of milrinone in conjunction with diuretics for 6 days facilitated the weaning from dobutamine.

Echocardiography at admission showed a velocity-time integral (VTI) of 6 cm/s, improving to 12 cm/s the next day. There was global LV dysfunction on TTE with an LVEF of 35%. A TTE performed on Day 9 revealed a slightly dilated LV with dyskinesis, particularly on the lateral wall, and a shortening fraction of 25%. Subsequent checks indicated normalization of LV function.

Troponin levels were at 80 on admission and peaked at 8670 ng/L on Day 3 and then decreased. Peak creatine phosphokinase (CPK) reached 3120 on Day 4. Her recovery was complicated by two failed embolizations and subsequent left hemiplegia with complex oculomotor disorders. After a year, she experienced a prolonged coma due to a recurrence of AVM rupture, resulting in severe neurological disabilities.

### Patient 3

A 16-year-old Caucasian girl, weighing 60 kg with a history of mitral insufficiency due to prolapse, was admitted after experiencing a cardiocirculatory arrest with ventricular fibrillation. She was revived after receiving two electric shocks and a dose of adrenaline. Upon admission, her Glasgow Coma Scale score was 7.

She required haemodynamic support with adrenaline at 0.3 µg/kg/min and noradrenaline up to 0.3 µg/kg/min, which was tapered off by Day 3. The initial TTE revealed a dilated LV and septal/apical/anterior hypokinesis, indicating cardiac stress disease. The LVEF was estimated at 31%. The subaortic VTI, bifid due to a septal defect, was measured at 9–10 cm, with an estimated cardiac output of 5.2 L/min.

The ECG showed a sinusoidal rhythm, early anterior repolarization, and large, sharp precordial T-waves. Persistent stress cardiac features were observed on TTE, with systemic vascular resistance calculated at over 2500 dyn/m^2^. Troponin levels peaked at 4500 ng/L but improved alongside ultrasound findings. The TTE on Day 3 indicated significant improvement, with LVEF over 50%, normalized cardiac output, but continued septo-apical hypokinesia. Coronary angiography results were normal.

The aetiological investigation was inconclusive, except for a possible neurological cause indicated by a brain scan showing bifrontal oedema. The patient’s neurological and haemodynamic condition improved favourably.

### Patient 4

A 10-year-old Caucasian girl, weighing 30 kg and with no significant medical history, was admitted to the PICU for observation following the surgical removal of a posterior fossa tumour. About 24 h post-surgery, she developed refractory hypoxia, indicating cardiogenic shock, alongside ventricular shunt dysfunction. With sedation, her Glasgow Coma Scale score was 8.

Treatment with milrinone was initiated, reaching a maximum dosage of 0.61 µg/kg/min and gradually tapered off by Day 10. Additionally, norepinephrine (maximum dose 0.18 µg/kg/min) and dobutamine (maximum doses 12.2 µg/kg/min) were initially administered and weaned off by 48 h.

The initial TTE revealed myocardial dysfunction, characterized by a shortening fraction of 6%. The LV was notably hypokinetic and extremely dilated, with dilated pulmonary veins and an almost flat septum. The ECG displayed monomorphic premature ventricular complexes, but no rhythm disturbances or signs of myocardial injury were observed. Acute phase troponin levels peaked at 12 000 ng/L, and brain natriuretic peptide (BNP) levels reached 1488 ng/L. By Day 4, the TTE had normalized.

Her recovery was marked by haemodynamic stability and complete normalization of the TTE. However, her condition was complicated by the progression of medulloblastoma, which eventually metastasized.

### Patient 5

A 5-month-old Caucasian girl, weighing 8 kg and with a history of left brachial plexus issues, was admitted to the PICU for septic shock resulting from pneumococcal meningitis. Upon arrival, she was intubated and given dopamine to manage hypotension. With sedation, her Glasgow Coma Scale score was 3. Haemodynamic support initially involved dopamine and dobutamine, with noradrenaline added later and continued for 7 days. On the third day, milrinone replaced dobutamine.

The initial TTE revealed LV dysfunction, evidenced by a subaortic VTI of 7 cm/s. The ultrasound showed an unusual pattern with normal movement around the mitral annulus but near akinesis at the apex and interventricular septum. Her peak troponin level was 817 ng/L and gradually decreased. Clinical and haemodynamic management required several vascular fillings. A follow-up TTE on Day 8 indicated improved LV function, leading to a reduction and eventual cessation of milrinone on Day 9.

Her condition was further complicated by pneumococcal haemolytic uraemic syndrome and an increase in intracranial pressure. Despite intensive management, her neurological condition deteriorated, and she passed away 1 month later.

### Patient 6

A 9-year-old Caucasian girl, weighing 25 kg and with a history of eating disorders, was admitted to intensive care following a respiratory arrest caused by a cerebellar injury due to a high-grade glioma. Upon admission, she was vomiting but had a Glasgow Coma Scale score of 15. She underwent emergency surgery, which included the insertion of an external ventricular shunt and decompression of the posterior cerebral fossa. Initially, her haemodynamic status was maintained with noradrenaline, and she had stable ventricular function. However, she experienced a rapid decline in her condition due to stress cardiomyopathy, leading to a cardiorespiratory arrest (lasting <1 min) upon her arrival at the PICU. This required the administration of adrenaline, dobutamine, and hydrocortisone hemisuccinate. During the surgery, she developed significant haemodynamic instability, with adrenaline doses reaching up to 1.4 µg/kg/min and dobutamine up to 20 µg/kg/min. Her lactate levels spiked to 12 mmol/L immediately following the surgery. A TTE revealed a global dysfunction with a LVEF of 36%. Her troponin levels increased to 2475 ng/L. Afterward, she was stabilized on noradrenaline at 0.5 µg/kg/min and dobutamine at 20 µg/kg/min, and her lactate levels decreased to 2.4 mmol/L. Unfortunately, her condition rapidly deteriorated, ultimately leading to brain death.

## Discussion

In this report, we present six paediatric cases of stress cardiomyopathy treated in a PICU. Our findings show a higher occurrence in female children, deviating from earlier studies that indicated a more balanced distribution between male and female children.^11,12^ This contrasts with adult cases, predominantly female.^1^ It’s important to note that we’re limited to diagnosed cases; the condition is likely underdiagnosed, suggesting many cases may go unrecognized. The average age of onset was 9.4 years, aligning with previous paediatric studies. While adult mortality rates stand at about 4%^1^ and 7% in children, our study observed higher mortality rates. However, none of the deaths were directly linked to stress cardiomyopathy but rather to underlying diseases.

Diagnosing paediatric Takotsubo syndrome is challenging. Adult guidelines suggest excluding coronary artery disease, often through coronary angiography. But, due to the low likelihood of primary coronary involvement, this procedure is seldom performed in children. Alternatives like myocardial infarction in the absence of obstructive coronary artery disease (MINOCA) or low-flow myocardial ischaemia should be considered, especially in cases like cardiocirculatory arrest. Sepsis-related myocardial dysfunction is also a possibility. In our study, patient 5’s diagnosis of Takotsubo was made despite the dysfunction’s varied appearance on TTE. Nevertheless, each diagnosis requires careful consideration in paediatric medicine.

Interestingly, most of our stress cardiomyopathy cases stemmed from severe neurological issues, except one. Neurological distress is a known trigger for Takotsubo syndrome, as noted in the 2018 criteria.^9^ Takotsubo has been linked to neurological conditions like stroke, seizures, and subarachnoid haemorrhage. Autopsy studies have found frequent contraction band necrosis and involvement of the insular or posterior fossa regions,^16^ indicating a possible heart–brain link that merits further exploration. This connection might involve structural differences in the limbic network, including areas like the insula, cingulate cortex, amygdala, and hippocampus.^17^

Stress cardiomyopathy should be considered a significant factor in neurological diseases and actively monitored. The underlying mechanisms of stress cardiomyopathies remain elusive, but overstimulation of the sympathetic system, leading to excessive catecholamines, is thought to play a key role.^4,5^ The exact process by which this overstimulation causes transient LV dysfunction is debated. Theories range from impaired cardiac microvascularization to microvascular dysfunction, epicardial spasm, or direct adrenergic receptor–mediated myocyte injury. Another hypothesis involves catecholamine-induced cardiac myocyte damage, possibly through calcium overload. Hence, acute neurological conditions that elevate catecholamines could trigger Takotsubo.

An intriguing clinical question arises regarding the treatment of myocardial dysfunction caused by catecholamine excess with catecholamines. This approach could potentially worsen the syndrome. However, this issue is addressed in the recommendations.^9^ Managing shock in these cases is complex, both theoretically and practically. Non-aminergic measures, such as aortic counterpulsation or extracorporeal membrane oxygenation, are preferred. Levosimendan may also be beneficial. Furthermore, the blood concentration of catecholamines from infusions is likely much lower than levels observed during initial Takotsubo episodes. In some cases, catecholamine use may have improved neurological outcomes by enhancing cerebral perfusion in shock. It’s important to note that the adrenergic pathophysiological hypothesis of Takotsubo focuses on overstimulation of adrenergic receptors. However, the use of noradrenaline stimulates specific alpha receptors, primarily affecting vascular rather than myocardial actions, potentially leading to reperfusion scenarios.

An association between sepsis and Takotsubo has been noted in adult cases^18^ and some paediatric studies. The underlying mechanism remains uncertain but may involve decreased coronary perfusion causing myocardial ischaemia, the impact of inflammatory mediators like TNF-alpha or interleukin-1, sympathetic system stimulation leading to massive catecholamine release, or the administration of exogenous catecholamines.^19,20^ In our series, only one child had sepsis at diagnosis, but neurological triggers like pneumococcal meningitis were also observed.

The rarity and probable under-diagnosis of this condition in paediatrics might explain the discrepancies between our findings and existing literature. Conducting a multicentre study could provide deeper insights into the paediatric aspects of this condition. Currently, there’s no consensus on diagnosing and managing Takotsubo cardiomyopathy in children. Diagnostic criteria proposed by the Mayo Clinic^8^ are not universally accepted and may not be entirely applicable to children. However, Takotsubo in children can present similarly to adults in terms of triggers, ultrasound, biological, and electrocardiographic markers.

## Conclusion

Takotsubo syndrome can indeed affect the paediatric population. It’s important to consider this condition in any child who shows signs of haemodynamic instability, particularly following an acute neurological event. In such cases, conducting a transthoracic ultrasound promptly is crucial for initiating appropriate treatment. This condition generally has a favourable prognosis when diagnosed and treated quickly.

## Data Availability

Data available on request.

## Lead author biography

N.A. is a paediatrician, with an interest in emergency, intensive care, echography, and cardiology. She has a university diploma in congenital heart diseases in 2017, with a thesis in 2019. She worked in the neonatal intensive care in the Hospital University of Lyon. She works actually in paediatric emergency, in the Hospital University of Saint Etienne, since 2020 with an outside activity in the paediatric intensive care unit of the Mother and Child Hospital of Hospices Civils de Lyon since 2018.
